# Who Falls for Misinformation and Why?

**DOI:** 10.1177/01461672251328800

**Published:** 2025-03-31

**Authors:** Tyler J. Hubeny, Lea S. Nahon, Nyx L. Ng, Bertram Gawronski

**Affiliations:** 1 University of Texas at Austin, Austin, TX, USA

**Keywords:** misinformation, myside bias, personality, signal detection, truth judgment

## Abstract

Misinformation is widespread, but only some people accept the false information they encounter. This raises two questions: Who falls for misinformation, and why do they fall for misinformation? To address these questions, two studies investigated associations between 15 individual-difference dimensions and judgments of misinformation as true. Using Signal Detection Theory, the studies further investigated whether the obtained associations are driven by individual differences in truth sensitivity, acceptance threshold, or myside bias. For both political misinformation (Study 1) and misinformation about COVID-19 vaccines (Study 2), truth sensitivity was positively associated with cognitive reflection and actively open-minded thinking, and negatively associated with bullshit receptivity and conspiracy mentality. Although acceptance threshold and myside bias explained considerable variance in judgments of misinformation as true, neither showed robust associations with the measured individual-difference dimensions. The findings provide deeper insights into individual differences in misinformation susceptibility and uncover critical gaps in their scientific understanding.

Misinformation is a widespread part of 21st century life and its prevalence can present major problems for the functioning of societies. For example, a survey in the United States found that 73% of participants reported having encountered misinformation about COVID-19 vaccines, and that those who had been exposed to misinformation were more than 1.5 times less likely to be vaccinated (Neely et al., 2022). However, despite the vast majority having been exposed to misinformation about COVID-19 vaccines, less than 20% of the United States population is unvaccinated as of May 11, 2023 (Centers for Disease Control and Prevention, 2023). Evidently, only some people fall for the misinformation they encounter. This raises two questions: (a) who falls for misinformation, and (b) why do they fall for misinformation? The current research addressed these questions by investigating associations between various individual-difference dimensions and judgments of misinformation as true. Using Signal Detection Theory (SDT; Green & Swets, 1966), we identified three distinct factors underlying judgments of misinformation as true and examined the specific ways in which the measured individual-difference dimensions contribute to belief in misinformation. Because we were interested in general propensities to fall for misinformation, we investigated individual differences in misinformation susceptibility in two content domains (i.e., politics, COVID-19 vaccines) and focused on associations that replicated across both.

## Why Do People Fall for Misinformation?

To answer the question of whether certain individual-difference characteristics are associated with a stronger susceptibility to misinformation, it is important to first consider why people may fall for misinformation. SDT suggests three distinct factors that can lead people to mistakenly judge misinformation as true: (a) low truth sensitivity, (b) low acceptance threshold, and (c) myside bias (Batailler et al., 2022).

To illustrate these factors, consider the four cases implied by judgments of true and false information as either true or false. Using SDT terminology, judgments of true information as true can be described as *hits*; judgments of false information as true can be described as *false alarms*; judgments of true information as false can be described as *misses*; and judgments of false information as false can be described as *correct rejections* (see Batailler et al., 2022; Gawronski et al., 2023). From the perspective of SDT, the question of why people fall for misinformation can be restated as: Why do people judge false information as true? Or, in technical terms: Why do people show false alarms?

One factor that can lead people to mistakenly judge false information as true is that they may be unable to accurately distinguish between true and false information. In this case, people would show not only a high rate of false alarms but also a high rate of misses. Using SDT terminology, this case can be described as an instance of low truth sensitivity (Batailler et al., 2022; Gawronski et al., 2023). A second factor that can lead people to mistakenly judge false information as true is that they may tend to judge all information they encounter as true. In this case, people would show a high rate of hits in addition to showing a high rate of false alarms. Using SDT terminology, this case can be described as an instance of low acceptance threshold (Batailler et al., 2022; Gawronski et al., 2023). Finally, a third factor that can lead people to mistakenly judge false information as true is myside bias, defined as the tendency to evaluate information in a manner biased toward one’s personal opinions (Stanovich et al., 2013). When making judgments about true and false information, myside bias involves a tendency to accept information as true if it is congruent with one’s views and to dismiss information as false if it is incongruent with one’s views, irrespective of whether the information is true or false. In terms of SDT, this pattern is reflected in a lower acceptance threshold for information that is congruent with one’s views compared to information that is incongruent with one’s views (Batailler et al., 2022; Gawronski et al., 2023).

A central goal of the current work was to identify individual-difference dimensions associated with truth sensitivity, acceptance threshold, and myside bias. Although numerous prior studies have sought to identify individual-difference dimensions associated with misinformation susceptibility (e.g., Bronstein et al., 2019; Escolà-Gascón et al., 2023; Pennycook & Rand, 2020) and a growing body of work has used SDT to analyze belief in misinformation (e.g., Gawronski et al., 2023; Modirrousta-Galian & Higham, 2023; Nahon et al., 2024), the current work is the first to use SDT to systematically examine individual differences in truth sensitivity, acceptance threshold, and myside bias. While there are notable exceptions that have examined relationships with acceptance threshold in addition to truth sensitivity (Bronstein et al., 2019; Lin et al., 2023; Ludwig & Sommer, 2024), none of these studies included myside bias. Furthermore, all prior studies focused exclusively on individual-difference dimensions typically thought to be related to truth sensitivity (e.g., cognitive reflection, bullshit receptivity), leaving unexplored theoretically plausible individual-difference correlates of acceptance threshold and myside bias.

By taking a signal-detection approach, we address several limitations of prior work. First, by evaluating all three factors, we can assess whether and to what extent individual differences in each of the three factors uniquely contribute to misinformation susceptibility. Second, by examining individual-difference dimensions in relation to all three factors simultaneously, we can investigate the multiple routes through which an individual-difference dimension may relate to misinformation susceptibility. Identifying relationships with all three factors is particularly important for the development of person-centered interventions aimed at reducing misinformation susceptibility. For example, if an intervention aims to increase truth sensitivity, that intervention may be effective for people who fall for misinformation due to low truth sensitivity, but it would likely be ineffective for people who fall for misinformation due to low acceptance threshold or strong myside bias (Gawronski et al., 2024). Thus, understanding the nuanced relationships with all three factors is essential for providing a complete picture of why people with certain individual-difference characteristics fall for misinformation. Finally, while previous studies have largely examined individual differences in misinformation susceptibility in a single content domain, it is possible that the obtained results are limited to that content domain. We address this limitation by investigating individual differences in misinformation susceptibility in two distinct content domains (i.e., politics, COVID-19 vaccines), focusing specifically on associations that replicate across content domains.

## Individual Differences in Misinformation Susceptibility

We relied on extant theoretical accounts of truth sensitivity, acceptance threshold, and myside bias to identify several individual-difference dimensions likely to be related to the three factors. In the following sections, we briefly discuss these accounts and their implications for various individual-difference dimensions that may contribute to misinformation susceptibility. We also review prior empirical work on individual differences in misinformation susceptibility and discuss potential relations of the studied individual-difference dimensions to the three factors underlying belief in misinformation.

### Truth Sensitivity

One frequently cited explanation for belief in misinformation is lack of analytic reasoning (Pennycook & Rand, 2021). According to this account, people with an analytic (vs. intuitive) thinking style should be less prone to believing misinformation. Consistent with this account, studies across many countries have found that people with higher scores on the Cognitive Reflection Test (CRT; Frederick, 2005) show greater accuracy in discerning real news from fake news (e.g., Arechar et al., 2023; Pennycook & Rand, 2019, 2020). Another individual-difference dimension linked to the analytic-reasoning account is actively open-minded thinking (AOT), a thinking style that encompasses reflectiveness, tolerance for ambiguity, and a tendency to embrace new information that disconfirms favored beliefs (Stanovich & Toplak, 2023). Although AOT was originally conceived of as an antidote to myside bias, AOT has been found to be positively related to analytic reasoning as well as truth discernment in judgments of real and fake news (e.g., Bronstein et al., 2019; Mirhoseini et al., 2023; Roozenbeek et al., 2022). We expected to replicate these findings within an SDT framework, in that individual differences in cognitive reflection and AOT should be positively associated with truth sensitivity in judgments of true and false information (Batailler et al., 2022).

Beyond testing individual differences related to the analytic-reasoning account, we also included a measure of the Big-5 to explore the role of basic personality traits. Available evidence for associations between Big Five personality traits and misinformation susceptibility is mixed (Calvillo et al., 2024). For example, while Calvillo et al. (2021) found fake-news discernment to be positively associated with agreeableness, conscientiousness, and openness, results from Sindermann et al. (2021) found only a negative association with extraversion. Additionally, research by Bainbridge et al. (2019) found associations between Big Five personality traits and receptivity to pseudo-profound bullshit, but their study did not examine associations with discernment of true and false information. Finally, while work by Piksa et al. (2023) did investigate relations between fake-news susceptibility and Big Five personality factors, their methodological and analytic design does not allow for adequate extrapolations to an SDT approach. As such, the current body of evidence is insufficient to clearly determine whether Big Five personality factors are meaningfully associated with truth sensitivity.

A major limitation of prior work on individual differences in misinformation susceptibility is that it focused almost exclusively on people’s ability to discern true from false information (i.e., truth sensitivity), providing no information on individual differences in acceptance threshold and myside bias. Yet, to fully understand an individual-difference dimension’s role in misinformation susceptibility, it is essential to examine its relationship to all three factors that contribute to the acceptance of misinformation. In the current work, we aimed to address this limitation by investigating individual-difference correlates of all three factors.

### Acceptance Threshold

Prior work on truth judgments suggests that people have a relatively low acceptance threshold overall, reflecting the fact that most information people encounter is true (Brashier & Marsh, 2020). Nevertheless, people may systematically differ in their tendency to accept information as true. One relevant factor in this regard might be receptivity to pseudo-profound bullshit (henceforth, bullshit receptivity), which refers to the tendency to judge nonsensical statements as deep and profound (Pennycook et al., 2015). Although bullshit receptivity has been linked to greater intuitive thinking and poorer fake-news discernment (Pennycook & Rand, 2020), recent work using SDT found bullshit receptivity to be associated with acceptance thresholds and not truth sensitivity (Ludwig & Sommer, 2024). Based on these considerations, we expected bullshit receptivity to be negatively associated with acceptance threshold.

Another potential correlate of acceptance threshold is the Big-5 dimension of conscientiousness. In studies on misinformation sharing, participants low in conscientiousness have been found to show a lower threshold for sharing information in general, regardless of its veracity (Lin et al., 2023). Although sharing decisions involve different processes and goals than truth judgments (Gawronski et al., 2023), individuals high in conscientiousness may generally be more cautious in their judgments and show a heightened sense of responsibility regarding the negative impacts of misinformation. Based on these considerations, we tested whether conscientiousness is positively associated with acceptance thresholds in truth judgments.

### Myside Bias

One prominent account to explain myside bias in judgments of misinformation identifies motivated reasoning as a driving force (see Kruglanski et al., 2020; Kunda, 1990). A central assumption of this account is that having cherished beliefs affirmed feels good, whereas having cherished beliefs threatened feels bad. Thus, based on the assumption that people who feel good about themselves have a weaker need to regulate their self-feelings by protecting their cherished beliefs, we tested the hypothesis that high self-esteem would be associated with weaker myside bias. Conversely, we tested whether people with high levels of neuroticism (also called negative emotionality) would show greater myside bias, due to a heightened motivation to mitigate negative emotions. Beyond motivations related to cherished beliefs, people are also motivated to protect their social identities (Van Bavel et al., 2024). Following this line of reasoning, we tested whether myside bias is more pronounced among people with a high need to belong (Leary et al., 2013) as well as those who strongly identify with likeminded others (McFarland et al., 2012). Another individual-difference dimension related to motivated reasoning is conspiracy beliefs (Krekó, 2015). Although often studied in relation to fake news discernment (e.g., Čavojová et al., 2024), conspiracy mentality—the tendency to appeal to secret plots and conspiracies (Bruder et al., 2013)—has been identified as a central factor underlying motivated reasoning in judgments of scientific evidence (Hornsey & Fielding, 2017). Thus, based on the presumed link to motivated reasoning, we tested whether conspiracy mentality is related to stronger myside bias.

An alternative account of myside bias in judgments of misinformation refers to the notion of Bayesian inference, suggesting that myside bias is the product of strongly held beliefs (or strong Bayesian priors) rather than motivated reasoning (Pennycook & Rand, 2021). According to this account, individual-difference dimensions related to the strength of one’s prior beliefs should be associated with myside bias. One such individual-difference dimension is grandiose narcissism. Although grandiose narcissism has been linked to misinformation susceptibility via a lack of analytic reasoning (Littrell et al., 2020, 2024), a central characteristic of grandiose narcissism is overconfidence (Macenczak et al., 2016). We thus tested the hypothesis that grandiose narcissism would be related to increased myside bias (see Enders et al., 2023; Escolà-Gascón et al., 2023). Another individual-difference dimension related to strong prior beliefs is the need to evaluate, defined as the tendency to engage in evaluative responding (Jarvis & Petty, 1996). Expanding on the idea that individuals with a greater need to evaluate have a stronger tendency to take a stance, we expected the need to evaluate to be associated with strong prior beliefs, and hence, increased myside bias.

On the flip side, a Bayesian account of myside bias would suggest that individual-difference dimensions associated with weaker prior beliefs should be related to lower myside bias. One individual-difference construct closely related to this idea is intellectual humility—defined as the degree to which people recognize that their beliefs might be wrong (Leary et al., 2017). Expanding on this definition, a Bayesian account would suggest that intellectual humility should be negatively related to myside bias (see Bowes et al., 2022). Similarly, AOT is likely related to weaker prior beliefs, given the tendency to embrace new information that disconfirms favored beliefs (Stanovich & Toplak, 2023). Although AOT has consistently failed to relate to other instances of myside bias (e.g., Macpherson & Stanovich, 2007; Stanovich & West, 2007, 2008; Toplak & Stanovich, 2003), some have continued to argue for AOT’s significance as an antidote to myside bias (Baron et al., 2015; Baron, 2024; Roozenbeek et al., 2022). Because of AOT’s conceptual relation to the Bayesian account of myside bias, and because we utilize a different operationalization of myside bias than previous studies, we hypothesized that AOT is related to lower myside bias. Finally, another factor that may be associated with weaker prior beliefs is the Big-5 measure of openness. Because intellectual curiosity is a key feature of openness, we expected openness to be related to reduced myside bias.

## The Current Research

The overarching goal of the current research was to provide deeper insights into individual-difference dimensions associated with misinformation susceptibility. Using SDT to quantify individual differences in truth sensitivity, acceptance threshold, and myside bias, we investigated (a) whether certain individual-difference characteristics make people more likely to fall for misinformation and (b) why a given individual-difference characteristic makes people more likely to fall for misinformation. To this end, we first examined the extent to which individual differences in truth sensitivity, acceptance threshold, and myside bias predict judgments of misinformation as true. In a second step, we investigated associations between 15 individual-difference measures and judgments of misinformation as true. Finally, in a third step, we tested whether the associations identified in the second step are driven by individual differences in truth sensitivity, acceptance threshold, or myside bias.

Together, the findings of the three steps go beyond prior work by providing more nuanced insights into individual differences in misinformation beliefs. The findings of the first step provide insights into the extent to which individual differences in truth sensitivity, acceptance threshold, and myside bias account for individual differences in judgments of false information as true. The findings of the second step provide insights into individual-difference characteristics that make people more likely to fall for misinformation. Finally, the findings of the third step provide insights into why a given individual-difference characteristic makes people more likely to fall for misinformation. By investigating individual differences in misinformation susceptibility in two content domains (i.e., politics, COVID-19 vaccines) and by focusing on associations that replicate across both, our findings also provide more compelling evidence for general propensities to fall for misinformation compared to prior studies that focused on a single content domain.

To accomplish these objectives, participants in the current studies were asked to complete measures capturing the Big-5, cognitive reflection, AOT, bullshit receptivity, self-esteem, need to belong, identification with likeminded people, grandiose narcissism, need to evaluate, intellectual humility, and conspiracy mentality. Next, participants completed a misinformation task, in which they made truth judgments about a series of statements. In Study 1, Democrats and Republicans judged true and false political statements that had either a pro-Democrat or pro-Republican slant. In Study 2, participants with favorable or unfavorable attitudes toward COVID-19 vaccines judged true and false statements about COVID-19 vaccines that had either a pro-vaccine or anti-vaccine slant.

## Open Practices

For each study, we report how we determined our sample size and all data exclusions, manipulations, and measures. The data, analysis codes, and research materials of both studies are available at https://osf.io/djprv/. The design, hypotheses, and analysis plans were preregistered for both studies. The preregistrations for Studies 1 and 2 are available at https://osf.io/vakj5 and https://osf.io/2v46n, respectively.

## Study 1

Study 1 investigated associations between individual-difference dimensions and susceptibility to political misinformation. In a first step, we tested the preregistered hypotheses that (a) judgments of ideology-congruent misinformation as true will be negatively associated with truth sensitivity and acceptance threshold, and positively associated with myside bias; and (b) judgments of ideology-incongruent misinformation as true will be negatively associated with truth sensitivity, acceptance threshold, and myside bias.

In a second step, we tested the preregistered hypotheses that judgments of misinformation as true will be (a) positively associated with bullshit receptivity, neuroticism, need to belong, identification with likeminded people, grandiose narcissism, need to evaluate, and conspiracy mentality, and (b) negatively associated with cognitive reflection, AOT, conscientiousness, self-esteem, intellectual humility, and openness.

In a third step, we tested the preregistered hypotheses that (a) truth sensitivity will be positively associated with cognitive reflection and AOT; (b) acceptance threshold will be negatively associated with bullshit receptivity and conscientiousness; and (c) myside bias will be positively associated with neuroticism, need to belong, identification with likeminded people, grandiose narcissism, need to evaluate, and conspiracy mentality, and negatively associated with self-esteem, intellectual humility, AOT, and openness.

### Method

#### Participants

Based on findings by Schönbrodt and Perugini (2013) suggesting that correlations in typical scenarios stabilize at around *N* = 250, we aimed to have a final sample of 250 participants after exclusions. Anticipating that approximately 15% of participants would be excluded based on preregistered exclusion criteria (see below), we recruited a total of 300 participants.

Participants were recruited on Prolific Academic. Participation was restricted to Prolific workers who (a) are fluent in English, (b) have an approval rate of at least 95% on prior assignments, (c) have completed at least 20 prior assignments, (d) currently reside in the United States, (e) are a citizen of the United States, and (f) did not participate in previous studies from our lab that used the same materials. To obtain a balanced sample, we recruited 150 Democrats and 150 Republicans using Prolific’s prescreening filter. In line with our preregistered exclusion criteria, participants were excluded from analyses if they failed an attention check or reported a political affiliation that was inconsistent with the one they had reported in Prolific’s prescreening survey. The study lasted approximately 30 min and participants were compensated US-$6.00.

Of the 314 participants who started the study, 300 completed all measures. Of these, 21 failed the attention check and an additional five reported inconsistent political affiliations. Data from these participants were excluded, leaving us with a final sample of 274 participants (141 Democrats, 133 Republicans). Demographic information on the final sample is provided in Table 1.

**Table 1.** Demographics.

Characteristic	Study 1	Study 2
Age—*M* (*SD*)	40.14 (13.05)	44.54 (14.60)
Nationality		
US	274	32
UK	—	190
Gender		
Female	139	116
Male	141	104
Prefer Not to Say	2	1
Other	2	1
Race		
Asian	18	9
Black or African American	43	8
White	194	201
Other	4	2
Multiple Races	15	2
Education		
Less Than High School Diploma	1	10
High School Diploma	37	86
Some College But No Degree*	60	—
Associate or Bachelor’s Degree	139	101
Master’s Degree	31	22
Doctoral Degree	6	3

*Note.* *The response option *Some college but no degree* was included only in Study 1.

#### Procedure and Measures

Participants completed a battery of 15 individual-difference measures in the order listed below, followed by a misinformation task, a demographic survey, and an attention check.

##### Big-5

Participants completed the 30-item Big Five Inventory-2 (Soto & John, 2017), including subscales for extraversion, agreeableness, conscientiousness, neuroticism, and openness. Responses were measured using 5-point rating scales ranging from 1 (*Disagree strongly*) to 5 (*Agree strongly*). After recoding reverse-coded items, mean scores were calculated for each subscale.

##### Need to Belong

Participants completed the 10-item Need to Belong Scale (Leary et al., 2013) which includes items such as *I want other people to accept me.* Responses were measured using 5-point rating scales ranging from 1 (*Not at all*) to 5 (*Extremely*). After recoding reverse-coded items, we computed the mean across all items.

##### Cognitive Reflection Test

Participants completed the original 3-item CRT (Frederick, 2005) and 4-item non-numeric version of the CRT (Thomson & Oppenheimer, 2016). The non-numeric version is recommended due to cognitive reflection being confounded with numeracy on the original CRT, which only uses items requiring mathematical abilities (Sinayev & Peters, 2015). The measure includes open-ended questions such as *A bat and a ball cost $1.10 in total. The bat costs $1.00 more than the ball. How much does the ball cost?*, which have an intuitive but incorrect answer (*10 cents*) and a non-intuitive but correct answer (*5 cents*). Scores were calculated as the total number of correct responses across all seven items, with correct answers coded as 1 and incorrect answers coded as 0.

##### Bullshit Receptivity

Participants completed the 10 items of Pennycook et al.’s (2015) bullshit receptivity measure. They rated the profoundness of randomly generated pseudo-profound statements (e.g., *Hidden meaning transforms unparalleled abstract beauty.*) using 5-point rating scales ranging from 1 (*Not at all profound*) to 5 (*Very profound*). Scores were calculated by computing the mean across all items.

##### Conspiracy Mentality

Participants completed the 5-item Conspiracy Mentality Questionnaire (Bruder et al., 2013). They rated the degree to which they agreed with generic conspiratorial statements such as *There are secret organizations that greatly influence political decisions*. Responses were measured with 11-point rating scales ranging from 0 (*0%—certainly not*) to 10 (*100%—certain*). Scores were calculated by computing the mean across all items.

##### Self-Esteem

Participants completed the 10-item Rosenberg Self-Esteem Scale (Rosenberg, 1965). They rated the degree to which they agreed with statements such as *I feel that I am a person of worth, at least on an equal basis with others.* Responses were measured with 4-point rating scales ranging from 1 (*Strongly disagree*) to 4 (*Strongly agree*). After recoding reverse-coded items, scores were calculated by computing the mean across all items.

##### Grandiose Narcissism

Participants completed the 16-item Narcissistic Personality Inventory (Ames et al., 2006), a measure of grandiose narcissism. Participants were presented with 16 sets of statements that had a narcissistic variant (e.g., *I think I am a special person*) and non-narcissistic variant (e.g., *I am no better nor worse than most people*). For each pair of narcissistic and non-narcissistic statements, participants chose the statement that best represented them. Narcissistic and non-narcissistic responses were coded as 1 and 0, respectively. Scores were summed across all items.

##### Intellectual Humility

Participants completed a 6-item measure of intellectual humility (Leary et al., 2017). They responded to items such as *I question my own opinions, positions, and viewpoints because they could be wrong* on 5-point rating scales ranging from 1 (*Not like me at all*) to 5 (*Very much like me*). Scores were calculated by computing the mean across all items.

##### Actively Open-Minded Thinking

Participants completed a 13-item measure of AOT (Stanovich & Toplak, 2023). They responded to items such as *People should always take into consideration evidence that goes against their opinions* on 6-point rating scales ranging from 1 (*Disagree strongly*) to 6 (*Agree strongly*). After recoding reverse-coded items, scores were calculated by computing the mean across all items.

##### Need to Evaluate

Participants completed the 16-item Need to Evaluate Scale (Jarvis & Petty, 1996). They responded to items such as *I form opinions about everything* on 5-point rating scales ranging from 1 (*Extremely uncharacteristic*) to 5 (*Extremely characteristic*). After recoding reverse-coded items, scores were calculated by computing the mean across all items.

##### Identification with Likeminded People

To measure the degree to which participants identify with likeminded people, we included an adapted version of the Identification with All Humanity Scale (McFarland et al., 2012). Participants responded to 10 questions such as *How close do you feel to each of the following groups?* for two groups, respectively. To target identification with others based on shared views, we included as a first group *people who share my views*. To isolate identification with likeminded people, we included as a second group *all humanity* and calculated the difference between *people who share my views* and *all humanity* ratings for each of the 10 items. Scores were then calculated by computing the mean across the resulting difference scores.

##### Misinformation Task

Participants read 80 news headlines. For each headline, they answered the following question *To the best of your knowledge, is the claim in this headline true or false?* using the response options *true* and *false*. The headlines varied in terms of whether the statement (a) was true or false and (b) had a pro-Democrat or pro-Republican slant (20 headlines per category). Pro-Democrat headlines were treated as ideology-congruent for Democrats and ideology-incongruent for Republicans, and vice versa for pro-Republican headlines. The headlines were gathered from the internet, screened, and pilot-tested (for details on the headline selection, see Supplemental Materials). The headlines were presented in a fixed random order as simple black text on a white background.

##### Demographics

Following the misinformation task, participants completed a series of demographic questions. First, participants rated their general, economic, and social political orientations. Next, participants reported their party affiliation with the response options *Democrat* and *Republican* and answered questions about their interest in politics, social media use, gender, age, race, ethnicity, education, and income.

##### Attention Check

A reading-intensive item was used as an attention check (Oppenheimer et al., 2009). In this attention check, participants read a lengthy paragraph that included instructions to provide no response to the item. Participants were deemed to have failed the attention check and excluded from analyses if they selected any of the response options.

#### Data Aggregation

Following our preregistered data aggregation plan, we first calculated hit and false-alarm rates for each participant. Hit rates (H) were calculated as the proportion of true news headlines judged as true; false-alarm rates (FA) were calculated as the proportion of false news headlines judged as true. Hit and FA rates were calculated for (a) ideology-congruent headlines, (b) ideology-incongruent headlines, and (c) all headlines. In cases where the proportion of *true* responses within a category was 0, we followed recommendations by Macmillan and Creelman (2004) and converted values to 1/(2 × *N*), with *N* being the number of trials (i.e., 20 in our case). In cases where the proportion of *true* responses was 1, we converted values to 1 − 1/(2 × *N*). Truth-sensitivity scores were calculated using SDT’s equation for discrimination sensitivity: *dʹ* = *z*(H) – *z*(FA). Higher truth-sensitivity scores reflect greater accuracy in distinguishing between true and false information. Acceptance-threshold scores were calculated using SDT’s equation for response threshold: *c* = −0.5 × [*z*(H) + *z*(FA)]. Higher scores reflect a stronger tendency to reject (vs. accept) information. An index of myside bias was calculated as the difference between SDT’s *c* scores for ideology-congruent versus ideology-incongruent headlines. Higher myside bias scores reflect a lower threshold for accepting ideology-congruent compared to ideology-incongruent headlines.

### Results

Means and standard deviations for hit, FA, miss, and correct-rejection rates are provided in Table 2. Means, standard deviations, and reliability estimates for the SDT indices and individual-difference measures are provided in Table 3.^1^ Results of multiple-regression analyses predicting acceptance of misinformation from truth sensitivity, acceptance threshold, and myside bias are provided in Table 4. Correlations between acceptance of misinformation, SDT indices, and all individual-difference measures are provided in Table 5.

**Table 2.** Rates of Hits, False Alarms, Misses, and Correct Rejections.

	Study 1		Study 2	
Variable	*M*	*SD*	*M*	*SD*
Hits	0.46	0.20	0.66	0.14
False Alarms	0.28	0.15	0.24	0.12
Misses	0.54	0.20	0.34	0.14
Correct Rejections	0.72	.15	0.76	0.12

**Table 3.** Means, Standard Deviations, and Reliability of Measures, Study 1.

Variable	*M*	*SD*	Cronbach’s Alpha
SDT Indices			
Truth Sensitivity	0.52	0.49	.68
Acceptance Threshold	0.38	0.50	.91
Myside Bias	0.66	0.70	.75
Individual-Difference Measures			
Extraversion	2.86	0.91	.78
Agreeableness	3.88	0.80	.80
Conscientiousness	3.78	0.90	.84
Neuroticism	2.62	1.10	.89
Openness	3.89	0.82	.81
Cognitive Reflection	4.02	2.03	.77
Actively Open-Minded Thinking	4.63	0.73	.84
Intellectual Humility	3.95	0.73	.86
Need to Evaluate	3.05	0.67	.86
Bullshit Receptivity	2.61	0.87	.88
Conspiracy Mentality	6.12	2.06	.86
Self-Esteem	3.06	0.74	.94
Grandiose Narcissism	3.42	3.16	.79
Need to Belong	2.83	0.81	.86
Identification with Likeminded People	0.35	0.65	.85

**Table 4.** Results of Multiple-Regression Analyses Predicting Acceptance of Ideology-Congruent and Ideology-Incongruent Misinformation from Truth Sensitivity, Acceptance Threshold, and Myside Bias, Study 1.

	Even → Odd		Odd → Even	
Variable	β	*p*	β	*p*
Acceptance of Ideology-Congruent Misinformation				
Truth Sensitivity (*d′*)	−.27	<.001	−.14	<.01
Acceptance Threshold (*c*)	−.67	<.001	−.49	<.001
Myside Bias	.14	<.01	.37	<.001
Acceptance of Ideology-Incongruent Misinformation				
Truth Sensitivity (*d’*)	−.19	<.001	−.32	<.001
Acceptance Threshold (*c*)	−.55	<.001	−.64	<.001
Myside Bias	−.52	<.001	−.35	<.001

*Note.* Even → Odd = responses on even items predicting responses on odd items. Odd → Even = responses on odd items predicting responses on even items.

**Table 5.** Correlations Between Individual Difference Measures and False-Alarm Rate, Truth Sensitivity, Acceptance Threshold, and Myside Bias, Study 1.

Variable	False-Alarm Rate	Truth Sensitivity	Acceptance Threshold	Myside Bias
Extraversion	.07	−.10	−.01	−.16**
Agreeableness	−.16*	−.03	.16**	.00
Conscientiousness	−.09	−.05	.12*	−.06
Neuroticism	.10	−.02	−.09	.00
Openness	−.06	.03	.02	.19**
Cognitive Reflection	−.19**	.30***	.02	.11
Actively Open-Minded Thinking	−.31***	.28***	.15*	.13*
Intellectual Humility	−.17**	.10	.11	−.09
Need to Evaluate	.19**	−.02	−.17***	.09
Bullshit Receptivity	.29***	−.25***	−.14*	−.25***
Conspiracy Mentality	.31***	−.14*	−.23***	.01
Self-Esteem	−.04	.06	.01	−.02
Grandiose Narcissism	.22***	−.24***	−.07	−.07
Need to Belong	.07	−.13*	.00	−.13*
Identification with Likeminded People	.04	.00	−.04	.00

*Note.* *p < .05, **p < .01, ***p < .001.

#### Relations between Acceptance of Misinformation and SDT Indices

Following our preregistered analysis plan, we first analyzed the extent to which acceptance of misinformation is predicted by truth sensitivity, acceptance threshold, and myside bias. To this end, we conducted multiple-regression analyses using indices of truth sensitivity, acceptance threshold, and myside bias to simultaneously predict judgments of false information as true (i.e., FA rate). Because FA rates are used to calculate the three predictors, we ensured mathematical independence of predictors and outcome by calculating all scores separately for responses to odd-numbered headlines and responses to even-numbered headlines in our database (see Gawronski et al., 2023). In one set of analyses, we used responses to odd-numbered items for the predictor variables and responses to even-numbered items for the outcome, and vice-versa in a second set of analyses. Because myside bias should increase acceptance of ideology-congruent misinformation and decrease acceptance of ideology-incongruent misinformation (Gawronski et al., 2023), we conducted separate analyses for acceptance of ideology-congruent and acceptance of ideology-incongruent misinformation as outcomes.

Results of the multiple-regression analyses confirmed all preregistered hypotheses (see Table 4). Specifically, acceptance of ideology-congruent misinformation showed significant negative associations with truth sensitivity and acceptance threshold, and a significant positive association with myside bias, regardless of whether responses to even-numbered items were used to predict responses to odd-numbered items or vice versa. Likewise, acceptance of ideology-incongruent misinformation showed significant negative associations with truth sensitivity, acceptance threshold, and myside bias, regardless of whether responses to even-numbered items were used to predict responses to odd-numbered items or vice versa.

#### Relations between Acceptance of Misinformation and Individual-Difference Measures

Following our preregistered analysis plan, we next analyzed correlations between the 15 individual-difference measures and judgments of misinformation as true (see Table 5). Consistent with our preregistered hypotheses, acceptance of misinformation showed significant positive associations with bullshit receptivity, conspiracy mentality, grandiose narcissism, and need to evaluate, and significant negative associations with cognitive reflection, AOT, and intellectual humility. Counter to our preregistered hypotheses, acceptance of misinformation showed no significant associations with conscientiousness, neuroticism, openness, need to belong, self-esteem, and identification with likeminded people. Additionally, acceptance of misinformation showed an unexpected negative association acceptance of misinformation showed an unexpected negative association with agreeableness with agreeableness.

#### Relations between SDT Indices and Individual-Difference Measures

Following our preregistered analysis plan, in a third step, we analyzed correlations between the 15 individual-difference measures and the three SDT indices (see Table 5).

##### Truth Sensitivity

Consistent with our preregistered hypotheses, truth sensitivity showed significant positive associations with cognitive reflection and AOT. Unexpectedly, truth sensitivity also showed significant negative associations with bullshit receptivity, conspiracy mentality, grandiose narcissism, and need to belong.

##### Acceptance Threshold

Consistent with our preregistered hypotheses, acceptance threshold showed a significant positive association with conscientiousness and a significant negative association with bullshit receptivity. Unexpectedly, acceptance threshold also showed significant positive associations with agreeableness and AOT, and significant negative associations with conspiracy mentality and need to evaluate.

##### Myside Bias

Counter to our preregistered hypotheses, myside bias was not significantly associated with neuroticism, identification with likeminded people, grandiose narcissism, need to evaluate, conspiracy mentality, self-esteem, and intellectual humility. Moreover, counter to our preregistered hypotheses, myside bias showed significant positive (rather than negative) associations with AOT and openness, and a significant negative (rather than positive) association with need to belong. Unexpectedly, myside bias also showed significant negative associations with extraversion and bullshit receptivity.

#### Robustness Check

Because Democrats and Republicans differed significantly on some of the measures (see Supplemental Materials) and because we were interested in general associations independent of political views, we gauged the robustness of the reported associations by conducting non-preregistered partial-correlation analyses controlling for political affiliation (see Supplemental Materials). For acceptance of misinformation and truth sensitivity, all aforementioned findings replicated. For acceptance threshold, the association with AOT (*r* = .11, *p* = .064) and bullshit receptivity (*r* = −12, *p* = .053) became non-significant. For myside bias, the association with AOT (*r* = .05, *p* = .452) and need to belong (*r* = −.12, *p* = .059) became non-significant, and the association with intellectual humility became statistically significant (*r* = −.17, *p* = .004).

As an additional robustness check, we conducted non-preregistered alpha-corrected analyses to account for multiple testing in the crossing of the three SDT factors and the 15 individual-difference measures (see Supplemental Materials). All effects in the preregistered analyses replicated, the only exceptions being that need to belong and self-esteem were no longer significantly related to truth sensitivity, conscientiousness was no longer significantly related to acceptance threshold, and AOT was no longer significantly related to myside bias.

### Discussion

Study 1 revealed three sets of notable findings. First, individual differences in truth sensitivity, acceptance threshold, and myside bias all independently predicted judgments of misinformation as true. Second, acceptance of misinformation was systematically related to a broad range of individual-difference dimensions. Third, the identified individual-difference dimensions differed in terms of whether they shaped acceptance of misinformation via truth sensitivity, acceptance threshold, or myside bias. However, while the obtained associations confirmed several of our preregistered hypotheses, some of our preregistered hypotheses were disconfirmed; yet other significant associations were unexpected. While our robustness checks suggest several of the unexpected associations were spurious in that they were driven by differences between Democrats and Republicans, the reliability of the other unexpected associations remains unclear. Thus, to gain greater confidence in the obtained associations and test their domain-independence, Study 2 aimed to replicate the findings of Study 1 in a different content domain.

## Study 2

Study 2 investigated associations between individual-difference dimensions and susceptibility to misinformation about COVID-19 vaccines. All preregistered hypotheses in Study 2 were based on the results of Study 1. That is, we hypothesized that all significant associations in the main analyses of Study 1 would replicate in Study 2. We followed the same data-analytic approach as in Study 1.

### Method

#### Participants

Following the rationale of Study 1, we aimed for a sample of 300 participants. Participants were recruited on Prolific Academic. Participation eligibility requirements were identical to Study 1, the only difference being that we included participants from the United Kingdom in addition to participants from the United States. To obtain a balanced sample, we recruited 150 Prolific workers with favorable attitudes toward COVID-19 vaccines and 150 Prolific workers with unfavorable attitudes toward COVID-19 vaccines, using Prolific’s prescreening filters. As in Study 1, we excluded participants if they failed the attention check or if they reported a COVID-19 vaccine attitude that was inconsistent with the one they had reported in Prolific’s prescreening survey. The study took approximately 30 min to complete, and participants were compensated US-$6.00.

Of the 309 participants who started the study, 301 completed all measures.^2^ Of the 301 participants with complete data, 72 failed the attention check and an additional seven reported inconsistent COVID-19 vaccine attitudes.^3^ Data from these participants were excluded, leaving us with a final sample of 222 participants (122 with pro-vaccine attitudes, 100 with anti-vaccine attitudes). Demographic information on the final sample is provided in Table 1.

#### Procedure

The procedure and measures were identical to Study 1, except that the misinformation task included statements about COVID-19 vaccines. The statements were directly adapted from Nahon et al. (2024) and varied in terms of whether they (a) were true or false and (b) had a pro-vaccine or anti-vaccine slant (20 statements per category; for details on the pilot-testing of the statements, see Nahon et al., 2024). In addition to completing the demographic measures of Study 1, participants were asked to describe their attitudes toward COVID-19 vaccines using the response options *For (I feel positively about the vaccines)* and *Against (I feel negatively about the vaccines)*. They also responded to questions about their vaccination status and COVID-19 experience.

### Results

Means and standard deviations for hit, FA, miss, and correct-rejection rates are provided in Table 2. Means, standard deviations, and reliability estimates for the SDT indices and individual-difference measures are provided in Table 6. Results of the multiple-regression analyses predicting acceptance of misinformation from truth sensitivity, acceptance threshold, and myside bias are provided in Table 7. Correlations between acceptance of misinformation, SDT indices, and all individual-difference measures are provided in Table 8.

**Table 6.** Means, Standard Deviations, and Reliability of Measures, Study 2.

Variable	*M*	*SD*	Cronbach’s Alpha
SDT Indices			
Truth Sensitivity	1.21	0.69	.85
Acceptance Threshold	0.15	0.24	.71
Myside Bias	0.90	1.04	.70
Individual-Difference Measures			
Extraversion	2.82	0.87	.78
Agreeableness	3.88	0.78	.81
Conscientiousness	3.70	0.86	.84
Neuroticism	2.66	1.04	.90
Openness	3.72	0.80	.76
Cognitive Reflection	4.23	1.97	.76
Actively Open-Minded Thinking	4.52	0.58	.79
Intellectual Humility	3.94	0.64	.84
Need to Evaluate	2.98	0.70	.89
Bullshit Receptivity	2.52	0.87	.88
Conspiracy Mentality	6.51	2.03	.86
Self-Esteem	3.01	0.63	.92
Grandiose Narcissism	2.43	2.67	.77
Need to Belong	2.74	0.77	.86
Identification with Likeminded People	0.25	0.70	.88

**Table 7.** Results of Multiple-Regression Analyses Predicting Acceptance of Attitude-Congruent and Attitude-Incongruent Misinformation from Truth Sensitivity, Acceptance Threshold, and Myside Bias, Study 2.

	Even → Odd		Odd → Even	
Variable	β	*p*	β	*p*
Acceptance of Attitude-Congruent Misinformation				
Truth Sensitivity (*d’*)	−.30	<.001	−.58	<.001
Acceptance Threshold (*c*)	−.15	<.001	−.20	<.001
Myside Bias	.54	<.001	.23	<.001
Acceptance of Attitude-Incongruent Misinformation				
Truth Sensitivity (*d’*)	−.42	<.001	−.04	.49
Acceptance Threshold (*c*)	−.24	<.001	−.20	<.001
Myside Bias	−.62	<.001	−.48	<.001

*Note.* Even → Odd = responses on even items predicting responses on odd items. Odd → Even = responses on odd items predicting responses on even items.

**Table 8.** Correlations Between Individual Difference Measures and False-Alarm Rate, Truth Sensitivity, Acceptance Threshold, and Myside Bias, Study 2.

Variable	False-Alarm Rate	Truth Sensitivity	Acceptance Threshold	Myside Bias
Extraversion	.07	−.11	.04	.12
Agreeableness	−.01	.08	−.07	−.09
Conscientiousness	.09	−.10	.00	.05
Neuroticism	.06	−.06	−.02	.01
Openness	.03	−.02	.02	.08
Cognitive Reflection	−.27***	.32***	−.01	−.12
Actively Open-Minded Thinking	−.36***	.37***	.08	−.12
Intellectual Humility	−.01	.07	−.08	−.08
Need to Evaluate	.23***	−.24***	−.03	.16*
Bullshit Receptivity	.24***	−.22***	−.07	−.02
Conspiracy Mentality	.39***	−.48***	.06	.26***
Self-Esteem	−.02	.00	.06	.07
Grandiose Narcissism	.19**	−.23***	.02	.16*
Need to Belong	−.10	.12	.00	−.16*
Identification with Likeminded People	.06	−.10	.04	.04

*Note.* *p < .05, **p < .01, ***p < .001.

#### Relations between Acceptance of Misinformation and SDT Indices

Following our preregistered analysis plan, we first conducted multiple-regression analyses using truth sensitivity, acceptance threshold, and myside bias to simultaneously predict judgments of false information as true (i.e., FA rate). Regression analyses were conducted separately for acceptance of attitude-congruent and acceptance of attitude-incongruent misinformation using the same odd-even split as in Study 1.

Results of the multiple-regression analyses confirmed our preregistered hypotheses for 11 of the 12 significance tests (see Table 7). Specifically, acceptance of attitude-congruent misinformation showed significant negative associations with truth sensitivity and acceptance threshold, and a significant positive association with myside bias, regardless of whether responses to even-numbered items were used to predict responses to odd-numbered items or vice versa. Acceptance of attitude-incongruent misinformation showed significant negative associations with acceptance threshold and myside bias, regardless of whether responses to even-numbered items were used to predict responses to odd-numbered items or vice versa. Truth sensitivity showed a significant negative association with acceptance of attitude-incongruent misinformation when responses to even-numbered items were used to predict responses to odd-numbered items, but this association was not statistically significant when responses to odd-numbered items were used to predict responses to even-numbered items.

#### Relations between Acceptance of Misinformation and Individual-Difference Measures

Following our preregistered analysis plan, in a second step, we analyzed correlations between the 15 individual-difference measures and judgments of misinformation as true (see Table 8). Consistent with the preregistered hypotheses based on the results of Study 1, acceptance of misinformation showed significant positive associations with bullshit receptivity, conspiracy mentality, grandiose narcissism, and need to evaluate, and significant negative associations with cognitive reflection and AOT. Counter to our preregistered hypotheses based on the results of Study 1, agreeableness and intellectual humility were not significantly associated with acceptance of misinformation.

#### Relations between SDT Indices and Individual-Difference Measures

Following our preregistered analysis plan, in a third step, we analyzed correlations between the 15 individual-difference measures and the three SDT indices (see Table 8).

##### Truth Sensitivity

Consistent with our preregistered hypotheses based on the results of Study 1, truth sensitivity showed significant positive associations with cognitive reflection and AOT, and significant negative associations with bullshit receptivity, conspiracy mentality, and grandiose narcissism. Unexpectedly, and different from Study 1, truth sensitivity also showed a significant negative association with need to evaluate. Counter to our preregistered hypothesis based on the results of Study 1, need to belong was not significantly associated with truth sensitivity.

##### Acceptance Threshold

Counter to our preregistered hypotheses based on the results of Study 1, acceptance threshold was not significantly associated with agreeableness, conscientiousness, AOT, bullshit receptivity, conspiracy mentality, and need to evaluate. Acceptance threshold also did not show significant associations with any of the other individual-difference measures.

##### Myside Bias

Consistent with the preregistered hypotheses based on the results of Study 1, myside bias was negatively associated with need to belong. Moreover, consistent with our initial hypotheses, but different from the results of Study 1, myside bias showed significant positive associations with need to evaluate, conspiracy mentality, and grandiose narcissism. However, counter to our preregistered hypotheses based on the results of Study 1, myside bias was not significantly associated with openness, extraversion, AOT, and bullshit receptivity.

#### Robustness Check

To rule out spurious correlations due to group differences, we again conducted non-preregistered partial-correlation analyses controlling for COVID-19 vaccine attitudes (see Supplemental Materials). For acceptance of misinformation, the association with grandiose narcissism became nonsignificant (*r* = .09, *p* = .207). For truth sensitivity, the association with grandiose narcissism (*r* = −.09, *p* = .207) and need to evaluate (*r* = −.13, *p* = .057) became nonsignificant. For acceptance threshold, the findings remained robust in that acceptance threshold did not show significant associations with any individual-difference measure. For myside bias, none of the significant associations were robust, in that need to belong (*r* = −.10, *p* = .124), need to evaluate (*r* = .03, *p* = .629), conspiracy mentality (*r* = −.05, *p* = .423), and grandiose narcissism (*r* = .00, *p* = .975) were not significantly associated with myside bias after controlling for vaccine attitudes. Bullshit receptivity, on the other hand, showed a significant negative association with myside bias after controlling for vaccine attitudes (*r* = −.15, *p* = .025).

As an additional robustness check, we again ran non-preregistered alpha-corrected analyses to account for multiple testing (see Supplemental Materials). All effects in the preregistered main analyses replicated in the alpha-corrected analyses.

### Discussion

Study 2 revealed three sets of notable findings. First, the results corroborate our finding that individual differences in truth sensitivity, acceptance threshold, and myside bias all independently contribute to judgments of misinformation as true. Second, we again found that acceptance of misinformation is systematically related to a broad range of individual-difference dimensions. Third, although the identified individual-difference dimensions differed in terms of how they shaped acceptance of misinformation, only associations with truth sensitivity replicated across the two studies. None of the 15 individual-difference dimensions showed reliable associations with acceptance threshold and myside bias across the two studies.

## General Discussion

The current research had two goals. First, we aimed to identify individual-difference dimensions associated with misinformation susceptibility. Second, we aimed to determine why people with certain individual-difference characteristics are more susceptible to misinformation: Is it because they show low truth sensitivity, low acceptance threshold, or strong myside bias? Across two studies that investigated susceptibility to misinformation about politics and COVID-19 vaccines, we found that cognitive reflection and AOT were associated with a weaker tendency to judge misinformation as true, whereas conspiracy mentality and bullshit receptivity were associated with a stronger tendency to judge misinformation as true. In all four cases, the obtained associations were driven by differences in truth sensitivity. Whereas cognitive reflection and AOT were associated with higher truth sensitivity, conspiracy mentality and bullshit receptivity were associated with lower truth sensitivity. We found no reliable associations for the Big-5, self-esteem, grandiose narcissism, need to belong, need to evaluate, intellectual humility, and identification with likeminded people. Notably, although truth sensitivity, acceptance threshold, and myside bias all independently predicted judgments of misinformation as true, we found no reliable associations between the 15 individual-difference measures and both acceptance threshold and myside bias.

### Truth Sensitivity

In both studies, individual differences in truth sensitivity explained considerable portions of variance in judgments of misinformation as true, with truth sensitivity being lower among participants low in cognitive reflection, participants low in AOT, participants high in conspiracy mentality, and participants high in bullshit receptivity. Although we did not originally hypothesize associations of bullshit receptivity and conspiracy mentality with truth sensitivity, the obtained associations are consistent with prior findings linking bullshit receptivity and conspiracy mentality to lack of analytic reasoning (e.g., Čavojová et al., 2024; Pennycook & Rand, 2020). One question that remains, though, is whether each of the four dimensions independently contributes to misinformation susceptibility, or whether they reflect different facets of a single unitary factor that drives the obtained associations with truth sensitivity. In line with the former idea, Roozenbeek et al. (2022) have argued that AOT and cognitive reflection relate to discernment ability via different psychological mechanisms. Yet, in line with the latter idea, factor-analytic findings by Pennycook and Rand (2020) suggest that cognitive reflection, bullshit receptivity, and fake-news discernment load onto a single unitary factor which they called *reflective open-mindedness*. However, the finding that the three variables load onto a single unitary factor does not imply that this unitary factor is the driving force behind the obtained associations with each specific variable. It also does not rule out the possibility that each individual variable contributes to truth sensitivity over and above the identified unitary factor.

**Figure 1.**
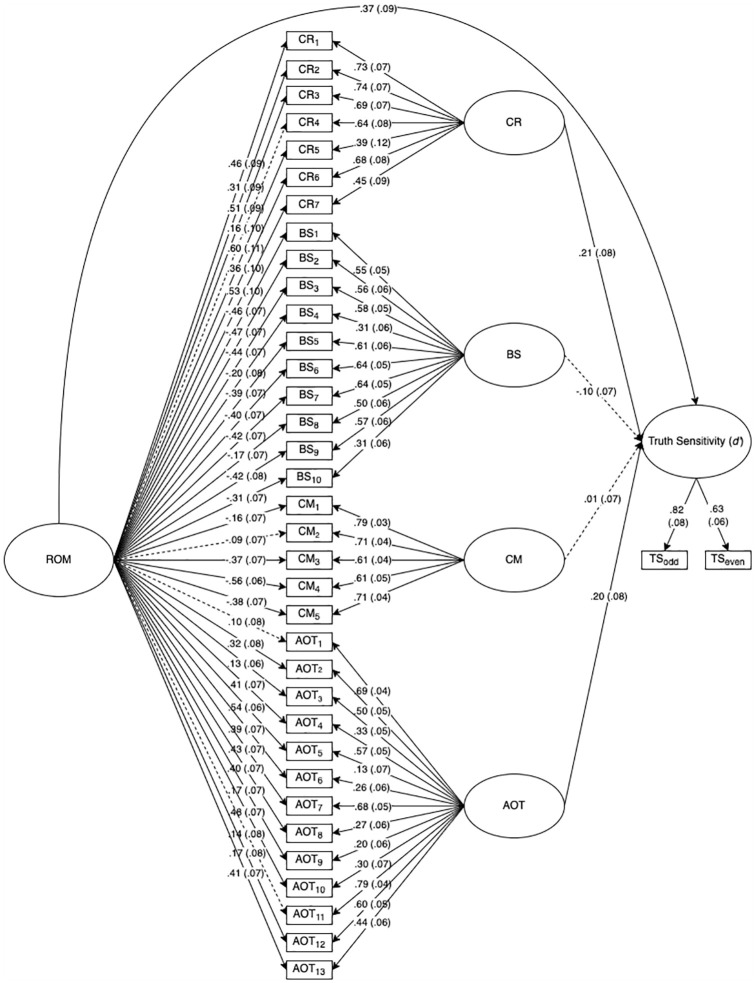
Results of bifactor structural equation model, Study 1. *Note.* ROM = Reflective Open-Mindedness; CR = Cognitive Reflection; BS = Bullshit Receptivity; CM = Conspiracy Mentality; AOT = Actively Open-Minded Thinking; TS_odd_ = Truth Sensitivity Scores calculated from odd-numbered items; TS_even_ = Truth Sensitivity Scores calculated from even-numbered items. Standardized estimates (standard errors) reported. Solid paths = significant (*p* < .05). Dotted paths = nonsignificant.

**Figure 2.**
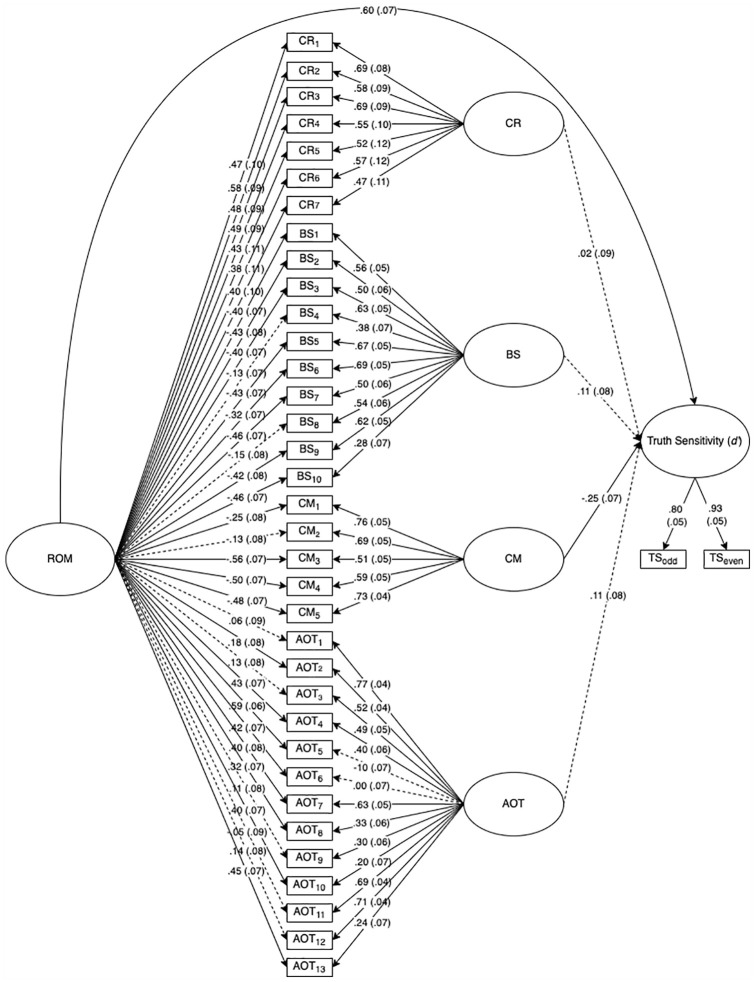
Results of bifactor structural equation model, Study 2. *Note.* ROM = Reflective Open-Mindedness; CR = Cognitive Reflection; BS = Bullshit Receptivity; CM = Conspiracy Mentality; AOT = Actively Open-Minded Thinking; TS_odd_ = Truth Sensitivity Scores calculated from odd-numbered items; TS_even_ = Truth Sensitivity scores calculated from even-numbered items. Standardized estimates (standard errors) reported. Solid paths = significant (*p* < .05). Dotted paths = nonsignificant.

A superior way to test these assumptions is through bifactor-model analysis (Rodriguez et al., 2016), which uses structural equation modeling to disentangle shared and unique variance of specific latent factors (e.g., cognitive reflection, AOT, conspiracy mentality, bullshit receptivity) and a general latent factor (e.g., reflective open-mindedness) with a given criterion (e.g., truth sensitivity). Figures 1 and 2 depict the results of non-preregistered bifactor model analyses of Studies 1 and 2’s data, respectively (for statistical details, see Supplemental Materials). The results suggest that truth sensitivity is reliably associated with a single general latent factor comprising cognitive reflection, AOT, bullshit receptivity, and conspiracy mentality. Following terminology proposed by Pennycook and Rand (2020), we call this general factor *reflective open-mindedness* (ROM). Independent of the general factor of ROM, the specific factors of cognitive reflection, AOT, and conspiracy mentality also showed significant associations with truth sensitivity over and above ROM. However, these associations did not replicate across studies, suggesting that they are either domain-specific or less reliable than the obtained association with ROM. Overall, these results suggest that the obtained associations of truth sensitivity with cognitive reflection, AOT, bullshit receptivity, and conspiracy mentality are largely driven by a single underlying factor.

### Acceptance Threshold

Like truth sensitivity, individual differences in acceptance thresholds explained considerable portions of variance in judgments of misinformation as true in both studies. Different from the results for truth sensitivity, however, individual differences in acceptance thresholds did not show reliable associations with any of the 15 individual-difference measures. These results suggest that individual differences in acceptance thresholds matter for misinformation susceptibility, but that either (a) individual differences in acceptance thresholds are domain-specific or (b) domain-independent differences in acceptance thresholds are related to individual-difference dimensions not measured in the current studies (or both). Overall, our findings indicate that individual differences in acceptance threshold are important for understanding individual differences in misinformation susceptibility, and they highlight the need for future research to identify individual-difference dimensions that are systematically related to individual differences in acceptance thresholds.

### Myside Bias

Like acceptance threshold, myside bias explained considerable portions of variance in judgments of misinformation as true in both studies, while not showing reliable associations with any of the 15 individual-difference measures. As with our conclusions for acceptance threshold, these results suggest that individual differences in myside bias matter for misinformation susceptibility, but that either (a) individual differences in myside bias are domain-specific or (b) domain-independent differences in myside bias are related to individual-difference dimensions not measured in the current studies (or both).

While future research may help to identify correlates of individual differences in myside bias, a notable aspect of the current findings is that greater thoughtfulness was not associated with lower myside bias: neither cognitive reflection nor AOT showed a significant negative association with myside bias (see also Batailler et al., 2022). These findings seem especially remarkable considering conceptualizations that define AOT as a “set of dispositions aimed at avoiding ‘myside bias’” (Baron et al., 2015, p. 267). Such a conceptualization conflicts not only with the current findings, but also with the results of several other studies (e.g., Macpherson & Stanovich, 2007; Stanovich & West, 2007, 2008; Toplak & Stanovich, 2003). To reconcile this paradox, Stanovich and Toplak (2023) suggested AOT may be related to lower myside bias only for weakly held beliefs but not for strongly held convictions—the latter of which may include the political beliefs (Study 1) and COVID-19 vaccine attitudes (Study 2) tested here. Regardless, while the current findings provide strong evidence that myside bias contributes to judgments of misinformation as true, the individual-difference characteristics associated with myside bias are still unclear, revealing a major gap in the field’s understanding of individual differences in misinformation susceptibility.

### Contributions

The current research contributes to the understanding of individual differences in misinformation susceptibility in several ways. First, our findings suggest that individual differences in truth sensitivity, acceptance threshold, and myside bias all contribute to individual differences in judgments of misinformation as true. While previous work has focused predominately on individual differences in truth sensitivity as the sole source of misinformation susceptibility (for notable exceptions, see Bronstein et al., 2019, Lin et al., 2023; Ludwig & Sommer, 2024), the current work demonstrates that all three factors must be considered to fully understand individual differences in misinformation susceptibility, further highlighting the value of SDT for research in this area.

Second, our findings support and extend prior work on truth discernment (e.g., Bronstein et al., 2019; Mirhoseini et al., 2023; Pennycook & Rand, 2020) by showing that individual differences in cognitive reflection, AOT, conspiracy mentality, and bullshit receptivity are associated with truth sensitivity via a single underlying factor. Yet, our findings go beyond supporting the analytic-reasoning account of truth discernment by also demonstrating that the four dimensions are not associated with acceptance threshold and myside bias. These null findings are notable because they suggest that, while analytic reasoning may reduce belief in misinformation by improving truth discernment, analytic reasoning is unlikely to reduce belief in misinformation when it is rooted in low acceptance threshold or myside bias.

Third, our findings highlight a major gap in the field’s understanding of individual differences in misinformation susceptibility. The fact that individual differences in acceptance thresholds and myside bias predicted acceptance of misinformation in both studies without showing reliable associations with any of the measured individual-difference dimensions demonstrates how focusing solely on truth sensitivity, without considering acceptance thresholds and myside bias, provides an incomplete understanding of individual differences in misinformation susceptibility. This result further highlights the value of applying SDT to the study of individual differences in misinformation susceptibility. The need for more research is particularly evident for myside bias, given that 10 of the 15 individual-difference measures included in the current studies were originally hypothesized to be associated with myside bias. Two reasons may account for these null results. On the one hand, it is possible that the reviewed accounts of myside bias are deficient in some way. On the other hand, it is possible that the reviewed accounts of myside bias are correct, but that the hypothesized links to the included individual-difference constructs are too weak. Although we cannot rule out the latter possibility, the fact that none of the included individual-difference measures were reliably associated with myside bias provides convergent evidence across multiple hypothesized links that both motivated-reasoning and Bayesian accounts may not fully capture individual differences in myside bias. Either way, the current findings demonstrate that, while the field has a clear understanding of individual differences in truth sensitivity, there is little to no understanding of individual differences in acceptance threshold and myside bias. Addressing this gap will be an important task for future research.

Fourth, by focusing on associations that replicate across content domains, our findings provide more compelling evidence for general propensities to fall for misinformation. Although associations between indicators of analytic reasoning and truth sensitivity have been replicated in various content areas and across many different countries, prior studies on other individual-difference correlates have focused predominantly on specific content domains without testing whether the obtained associations replicate in other content domains. The current findings suggest that many of the associations obtained in prior research are either not reliable or do not generalize across content domains, highlighting the importance of replicating findings on individual differences in misinformation susceptibility across different content domains.

Fifth, although not the focus of the current work, our findings raise the possibility that individual-difference correlates of acceptance threshold and myside bias differ across content domains. This possibility highlights the need for future work to focus on interactions between individual-difference dimensions and content domains to better elucidate the specific contexts in which certain individual-difference characteristics increase susceptibility to misinformation.

### Limitations and Future Directions

While the inclusion of distinct content domains permits stronger conclusions regarding general propensities to fall for misinformation, the reported findings were obtained with participants from two countries using two stimulus sets in two content domains, which raises the question of whether the obtained associations replicate in other samples, with other stimulus sets, and in other content domains. Moreover, both studies used the same set of individual-difference measures. While these measures have been extensively validated by prior research, it is possible that some of the obtained null effects are due to suboptimal characteristics of the employed measures rather than a genuine lack of associations with the to-be-measured constructs.

A related concern is that the current studies may have failed to replicate some earlier findings due to differences in the employed measures. For example, one could argue that differences in the employed measures of the Big-5 may account for some of the inconsistencies with prior findings. Relatedly, different from the measurement of binary truth judgments in the current studies, some prior studies used continuous measures of confidence in the perceived truth of a statement. However, while the difference between binary and continuous measures of truth judgments can impact results, any such differences have been found to be small and not meaningful (Roozenbeek et al., 2022). Nevertheless, future studies using different measures of the same construct may alleviate concerns about potential differences between measurement instruments.

While the current research focused on general propensities to fall for misinformation, an interesting question for future research is whether certain individual-difference dimensions are related to misinformation susceptibility in specific domains. Although we found several unique associations in one content domain but not the other, we caution against interpreting the current findings as evidence for content-dependent associations, because they could reflect (a) content-dependent associations, (b) false positives, or (c) false negatives. Because the three potential reasons for discrepant outcomes are indistinguishable based on the current data, more research is needed to address the question whether some individual-difference dimensions show content-dependent associations with misinformation susceptibility.

Finally, while the current studies included a larger number of theoretically-relevant individual-difference dimensions compared to previous studies, other characteristics likely matter as well. This is particularly evident for individual differences in acceptance threshold and myside bias, which explained substantial portions of variance in the acceptance of misinformation without showing reliable associations with any of the 15 individual-difference measures. As a first step toward addressing this issue, we conducted non-preregistered exploratory analyses investigating correlations of truth sensitivity, acceptance threshold, and myside bias with measures of political orientation, political interest, social media use, gender, age, and education (see Supplemental Materials). None of these measures showed reliable associations that replicated across the two studies.

## Conclusion

The main goal of the current research was to investigate (a) who falls for misinformation and (b) why do they fall for misinformation. Our findings suggest that people low in cognitive reflection, people low in AOT, people high in bullshit receptivity, and people high in conspiracy mentality are especially prone to judging misinformation as true—primarily because they are less able to distinguish between true and false information (i.e., truth sensitivity). Our findings further suggest that the obtained associations with the four individual-difference dimensions are related to a broader underlying construct that may be referred to as ROM. Moreover, while individual differences in truth sensitivity played a substantial role for judgments of misinformation as true, we also found evidence for systematic differences in acceptance threshold and myside bias, and these differences contributed to misinformation susceptibility over and above truth sensitivity. However, different from truth sensitivity, the individual-difference dimensions associated with acceptance threshold and myside bias remain unclear. Ultimately, this work highlights that, while the field has a relatively clear picture of individual differences in truth sensitivity, there is still much to be learned about individual differences in acceptance threshold and myside bias.

## Supplemental Material

sj-docx-1-psp-10.1177_01461672251328800 – Supplemental material for Who Falls for Misinformation and Why? Supplemental material, sj-docx-1-psp-10.1177_01461672251328800 for Who Falls for Misinformation and Why? by Tyler J. Hubeny, Lea S. Nahon, Nyx L. Ng and Bertram Gawronski in Personality and Social Psychology Bulletin

## Notes

1. Although *d’* and *c* are sensitive to violations of the assumptions of normality and constancy of variance, it is not possible to test violations of these assumptions in data from binary judgment tasks (Stanislaw & Todorov, 1999).

2. One participant completed all measures but did not submit a request for compensation.

3. One participant failed the attention check but messaged us stating they had read and understood the instructions but accidentally clicked on one of the responses. We marked this participant as having passed the attention check and included them in the final sample.
