# Supplementary material for: Who Falls for Misinformation and Why?
Source: Pers Soc Psychol Bull. 2025 Mar 31;52(7):1866–83. doi: 10.1177/01461672251328800 (PMC13216562; doi:10.1177/01461672251328800)
Supplement: sj-docx-1-psp-10.1177_01461672251328800 – Supplemental material for Who Falls for Misinformation and Why? [file sj-docx-1-psp-10.1177_01461672251328800.docx]

**Supplement:**

**Who Falls for Misinformation and Why?**

# Procedure for the Selection of Headlines Used in Study 1

## Headline Search

In a first step, a team of research assistants conducted a thorough search of mainstream-news and fact-checking websites, including AP News, BBC News, CNN, FactCheck.org, Fox News, LeadStories, New York Times, Snopes, Politico, Politifact, and Washington Post. In addition to following best practices for the selection of stimulus materials for research on misinformation (see Pennycook et al., 2021), the search was guided by the following criteria: (1) there should be agreement among Democrats and Republicans about whether a given headline has a pro-Democrat or a pro-Republican slant; (2) the focal issue should be directly related to one of the two parties instead of having a distal link to the two parties (e.g., no headlines about COVID-19 or Black Lives Matter); (3) the partisanship and evaluative connotation of the headlines should be directly evident from the statement and not depend on background assumptions that may differ across individuals; (4) the headlines should include statements about facts that can be true or false (rather than opinions); (5) the headlines must be unambiguously true or false instead of being misleading or having conflicting evidence regarding their truth; and (6) the headlines should not include source attributions (e.g., Person X said ABC), because those would render questions about their truth ambiguous (e.g., whether it is true that Person X said ABC or whether ABC is true). Three teams of research assistants regularly checked the identified websites during the academic years 2020-2021, 2021-2022, and 2022-2023, and added headlines that meet our inclusion criteria to a shared data base. For each headline added to the data base, the research assistants included the following information: (1) headline in its original wording; (2) veracity of the headline (i.e., true vs. false); (3) political leaning of the headline (i.e., pro-Democrat vs. pro-Republican); (4) original source of the headline; (5) publication date of the headline; (6) the date the headline was identified; (7) details on the story behind the headline; (8) information supporting the truth or falsity of the headline; (9) the initials of the person who added the headline to the data base, and (10) other notes on the headline.

## Initial Screening

In an initial screening of the identified headlines, we excluded all headlines that did not clearly meet our criteria, including headlines whose partisan slant might be perceived differently among Democrats and Republicans (e.g., headlines that may be perceived as pro-Democrat among Democrats, but as relatively neutral among Republicans); headlines that included a source attribution; and headlines whose veracity seemed ambiguous. We further excluded headlines whose content seemed too widely known; headlines that were outdated; headlines that relied too much on the context of the time at which it was published; headlines whose veracity could easily change with new developments; headlines that did not seem sufficiently partisan; and headlines whose wording seemed confusing.

## Pilot Testing

For each batch of headlines that passed our initial screening (*N* = 120 for batch 2020-2021; *N* = 105 for batch 2021-2022; *N* = 123 for batch 2022-2023), we conducted a pilot study with 120 self-identified Democrats and 120 self-identified Republicans via CloudResearch (Batch 2020-2021) or Prolific Academic (Batch 2021-2022 and Batch 2022-2023). In each of the three pilot studies, participants were asked two questions for each headline: (1) *How would you rate the political slant of this statement?* (2) *Have you heard about the claim in this statement before?* Responses to the first question were measured with 7-point rating scales with the endpoints *Very Pro-Democrat* (recorded as 1) and *Very Pro-Republican* (recorded as 7). Responses to the second question were measured with 7-point rating scales with the endpoints *Very confident I did not hear this before* (recorded as 1) and *Very confident I did hear this before* (recorded as 7). Based on the collected pilot data, we calculated the means and modes of partisanship scores and average familiarity scores for each headline. Scores were calculated separately for self-identified Democrats and self-identified Republicans. Based on the obtained scores, we eliminated headlines that were insufficiently partisan. To this end, we first excluded all pro-Democrat headlines with a mean partisanship score >3.00 among either Democrats or Republicans, and all pro-Republican headlines with a mean partisanship score <5.00 among either Democrats or Republicans. Next, we eliminated headlines with a mode partisanship score of 4 among either Democrats or Republicans. The 80 headlines used in Study 1 (20 pro-Democrat true; 20 pro-Republican true; 20 pro-Democrat false; 20 pro-Republican false) were selected from the three sets of headlines that passed the prescreening criteria in our pilot studies, using timeliness as an additional selection criterion. The final list of headlines used in Study 1 is available at <https://osf.io/djprv/?view_only=1038c3c0d5484629bb430c6f7b6f37e2>.

# Bifactor-Model Analyses

Across both studies, we found evidence for associations of truth sensitivity with individual differences in cognitive reflection, bullshit receptivity, conspiracy mentality, and actively open-minded thinking. Expanding on these findings, we tested whether truth sensitivity is predicted by (1) a general latent factor capturing the shared variance between all four individual-difference constructs, (2) specific latent factors for each of the individual-difference dimensions capturing the remaining unique variance amongst that specific construct not accounted for by the general factor, or (3) both. To this end, we utilized structural equation modeling to test a bifactor model (Rodriguez et al., 2016). Structural equation modeling allows us to estimate the associations of different latent factors with truth sensitivity by adding paths from each latent factor to truth sensitivity. Although factor analysis provides information on shared variance between items (see Pennycook & Rand, 2020), it does not test (a) whether the obtained associations are driven by a single unitary factor and (b) whether the specific individual factors predict truth sensitivity independently over and above its relation with a single unitary factor. To test the extent to which truth sensitivity is predicted by a general latent factor and/or specific latent factors, we ran a bifactor-model analysis in which all items of the four individual-difference measures were allowed to load onto (1) a general latent factor, which we call reflective open-mindedness (ROM), and (2) their respective specific latent factors. Thus, the general factor represents the shared variance across all items, whereas the specific factors represent the unique shared variance among items of a particular measure after controlling for the general factor.

The analyses were performed in R version 2.4.1 (R Core Team, 2022) using the *lavaan* package version 0.6.17 (Rosseel, 2012). In addition to estimating the general and specific latent factors as described above, we also estimated a latent factor of truth sensitivity from an odd-even split of the misinformation-task statements. To assess how much each factor contributes to truth sensitivity, we specified regression paths from ROM and each of the specific factors to truth sensitivity. Because items on the cognitive reflection test are binary (correct/incorrect), we utilized Weighted Least Squares Mean and Variance Adjusted (WLSMV) as the estimator, which is recommended when a mix of ordered and continuous data is used (Li, 2016). Items from all measures other than the cognitive reflection test were treated as continuous given that they all had five or more response options (Rhemtulla et al., 2012). All estimates reported are standardized.

Results showed that a bifactor model fit the data well in both Study 1, χ^2^(589) = 770.74, *p* < .001, RMSEA = .03, CFI = .91, SRMR = .06, and Study 2, χ^2^(589) =718.39, *p* < .001, RMSEA = .03, CFI = .92, SRMR = .07. To ensure that the general factor and specific factors are reliable and interpretable, we followed suggestions by Reise et al. (2023) and examined the explained common variance (ECV) and hierarchical omega (ω_H_) scores. These indices were calculated using the *BifactorIndicesCalculator* package version 0.2.2 (Dueber, 2021). ECV represents the degree to which the shared variance between items is due to the general factor. In both studies, the general factor explained about 30% of the common variance between items (Study 1: ECV = .31; Study 2: ECV = .32). Hierarchical omega scores represent the proportion of reliable variance in the composite score that can be explained by the general factor. High ω_H_ values (> .80) suggest that the items can be considered as essentially unidimensional. The ω_H_ values^[[1]](#footnote-2)^ for Study 1 and Study 2 were .58 and .60, respectively, suggesting that a significant proportion of the composite score could reliably be accounted for by the general factor, but not to a level that it could be considered as entirely unidimensional. Taken together, these results confirm the utility of the bifactor model, suggesting that the general factor of ROM is a reliable construct, but that the general factor alone cannot sufficiently account for the variance in the data.

Results of the structural equation model showed that, in both studies, the general latent factor of ROM significantly predicted truth sensitivity (Study 1 estimate = .37, *p* < .001; Study 2 estimate = .60, *p* < .001). Additionally, in Study 1, truth sensitivity was significantly predicted by the specific factors of cognitive reflection (estimate = .21, *p* = .009) and actively open-minded thinking (estimate = .20, *p* =.016), while in Study 2, truth sensitivity was significantly predicted by the specific factor of conspiracy mentality (estimate = -.25, *p* < .001). In Study 1, the specific factors of conspiracy mentality (estimate = -.01, *p* = .826) and bullshit receptivity (estimate = .10, *p* = .193) did not predict truth sensitivity, while in Study 2, the specific factors of cognitive reflection (estimate = .02, *p* = .822), actively open-minded thinking (estimate = .11, *p* = .175), and bullshit receptivity (estimate = .11, *p* = .154) did not predict truth sensitivity (see Figures 1 and 2 in the main article). Overall, these findings support the idea that there is a single, individual-difference factor that explains differences in truth sensitivity irrespective of the content domain. However, results of these bifactor models also suggest that there may be additional unique effects of different specific factors depending on the content domain.

**Alpha Correction**

While the decision to interpret significant associations only if they replicate across the two studies reduces the likelihood of false positives (Rubin, 2021), we also ran alpha-corrected analyses to ensure the robustness of the obtained results given the large number of tests. In these alpha-corrected analyses, we applied a Bonferroni correction to achieve an alpha level of .05 for the correlations between the 15 individual difference measures and the three SDT outcomes. Because we only interpret results that are significant in both Study 1 and Study 2 (i.e., conjunction test), the probability of a false-positive result is α^2^ (Rubin, 2021). Thus, given 45 tests and an alpha level of .05, the Bonferroni-corrected constituent alpha-level for each study can be calculated as $\sqrt{\frac{.05}{45}}= .033$. Therefore, we set our alpha level to .033.

After alpha correction in Study 1, need to belong and self-esteem were no longer significantly related to truth sensitivity, conscientiousness was no longer significantly related to acceptance threshold, and AOT was no longer significantly related to myside bias. There were no results that changed after alpha correction in Study 2. Examining only associations that replicate across both studies, alpha correction does not affect any of our conclusions.

# References

Dueber, D. (2021). *BifactorIndicesCalculator: Bifactor Indices Calculator*. R package version 0.2.2.

Li, C. H. (2016). Confirmatory factor analysis with ordinal data: Comparing robust maximum likelihood and diagonally weighted least squares. *Behavior Research Methods*, *48*, 936-949.

Pennycook, G., Binnendyk, J., Newton, C., & Rand, D. G. (2021). A practical guide to doing behavioral research on fake news and misinformation. Collabra: Psychology, *7*(1): Article 25293.

Pennycook, G., & Rand, D. G. (2020). Who falls for fake news? The roles of bullshit receptivity, overclaiming, familiarity, and analytic thinking. *Journal of Personality*, *88*(2), 185–200.

R Core Team (2022). *R: A language and environment for statistical computing*. R Foundation for Statistical Computing, Vienna, Austria.

Reise, S. P., Mansolf, M., & Haviland, M. G. (2023). Bifactor measurement models. In R. H. Hoyle (Ed.), *Handbook of structural equation modeling* (pp. 329-348). New York: Guilford Press.

Rhemtulla, M., Brosseau-Liard, P. É., & Savalei, V. (2012). When can categorical variables be treated as continuous? A comparison of robust continuous and categorical SEM estimation methods under suboptimal conditions. *Psychological Methods*, *17*(3), 354-373.

Rodriguez, A., Reise, S. P., & Haviland, M. G. (2016). Evaluating bifactor models: Calculating and interpreting statistical indices. Psychological Methods, 21(2), 137–150.

Rosseel, Y. (2012). lavaan: An R package for structural equation modeling. *Journal of Statistical Software*, *48*, 1-36.

Rubin, M. (2021). When to adjust alpha during multiple testing: A consideration of disjunction, conjunction, and individual testing. *Synthese*, *199*(3), 10969–11000.

**Table S1**

*Means and 95% Confidence Intervals of Measures for Democrats and Republicans, Study 1*

|  | Democrats | | Republicans | |
| --- | --- | --- | --- | --- |
|  | *n* = 141 | | *n* = 133 | |
| Variable | *M* | 95% CI | *M* | 95% CI |
| *Misinformation Indices* |  |  |  |  |
| False-Alarm Rate | 0.26 | [0.23, 0.28] | 0.31 | [0.28, 0.34] |
| Truth Sensitivity | 0.55 | [0.47, 0.62] | 0.49 | [0.40, 0.58] |
| Acceptance Threshold | 0.44 | [0.37, 0.50] | 0.32 | [0.22, 0.41] |
| Myside Bias | 0.82 | [0.72, 0.92] | 0.48 | [0.35, 0.61] |
| *Individual Difference Measures* |  |  |  |  |
| Extraversion | 2.74 | [2.58, 2.90] | 3.00 | [2.85, 3.14] |
| Agreeableness | 3.93 | [3.80, 4.06] | 3.84 | [3.69, 3.98] |
| Conscientiousness | 3.70 | [3.54, 3.86] | 3.86 | [3.71, 4.00] |
| Neuroticism | 2.73 | [2.53, 2.92] | 2.50 | [2.33, 2.68] |
| Openness | 4.06 | [3.93, 4.18] | 3.72 | [3.57, 3.86] |
| Cognitive Reflection | 4.33 | [4.00, 4.66] | 3.68 | [3.34, 4.03] |
| Actively Open-Minded Thinking | 4.88 | [4.77, 4.98] | 4.36 | [4.23, 4.49] |
| Intellectual Humility | 4.15 | [4.04, 4.25] | 3.73 | [3.60, 3.86] |
| Need to Evaluate | 3.11 | [2.99, 3.22] | 3.00 | [2.89, 3.10] |
| Bullshit Receptivity | 2.46 | [2.31, 2.62] | 2.76 | [2.62, 2.90] |
| Conspiracy Mentality | 5.67 | [5.34, 6.00] | 6.60 | [6.25, 6.95] |
| Self-Esteem | 3.01 | [2.88, 3.14] | 3.11 | [3.00, 3.23] |
| Grandiose Narcissism | 3.32 | [2.82, 3.82] | 3.53 | [2.97, 4.10] |
| Need to Belong | 2.77 | [2.63, 2.91] | 2.89 | [2.76, 3.02] |
| Identification with Likeminded People | 0.24 | [0.15, 0.34] | 0.46 | [0.34, 0.59] |

**Table S2**

*Correlations Between Individual-Difference Measures and Ideology-Congruent and Ideology-Incongruent False-Alarm Rates, Study 1*

| Variable | Ideology-Congruent False Alarm Rate | Ideology-Incongruent False Alarm Rate |
| --- | --- | --- |
| Extraversion | -.03 | .17** |
| Agreeableness | -.11 | -.14* |
| Conscientiousness | -.11 | -.01 |
| Neuroticism | .09 | .06 |
| Openness | .05 | -.17 |
| Cognitive Reflection | -.10 | -.20** |
| Actively Open-Minded Thinking | -.14* | -.37*** |
| Intellectual Humility | -.17** | -.09 |
| Need to Evaluate | .24*** | .05 |
| Bullshit Receptivity | .11 | .37** |
| Conspiracy Mentality | .22*** | .27*** |
| Self-Esteem | -.05 | -.01 |
| Grandiose Narcissism | .15* | .20*** |
| Need to Belong | .01 | .12 |
| Identification with Likeminded People | .02 | .05 |

Note. * p < .05, ** p < .01, *** p < .001

**Table S3**

*Correlations Between Individual-Difference Measures, Study 1*

| Variable | 1 | 2 | 3 | 4 | 5 | 6 |
| --- | --- | --- | --- | --- | --- | --- |
| 1. Extraversion | - |  |  |  |  |  |
| 2. Agreeableness | .31*** | - |  |  |  |  |
| 3. Conscientiousness | .43*** | .39*** | - |  |  |  |
| 4. Neuroticism | -.48*** | -.31*** | -.59*** | - |  |  |
| 5. Openness | .20*** | .21*** | .09 | -.09 | - |  |
| 6. Cognitive Reflection | -.18** | -.04 | -.11 | .00 | -.03 | - |
| 7. Actively Open-Minded Thinking | -.16** | .16** | .06 | -.03 | .20*** | .29*** |
| 8. Intellectual Humility | -.02 | .10 | -.07 | .00 | .21*** | .12* |
| 9. Need to Evaluate | .20*** | -.12* | -.02 | .03 | .22*** | -.14* |
| 10. Bullshit Receptivity | .22*** | .09 | .22*** | -.08 | -.05 | -.27*** |
| 11. Conspiracy Mentality | .06 | -.18** | -.08 | .09 | .01 | -.19** |
| 12. Self-Esteem | .52*** | .30*** | .52*** | -.71*** | .17** | -.04 |
| 13. Grandiose Narcissism | .49*** | -.11 | .10 | -.18** | .05 | -.15* |
| 14. Need to Belong | -.06 | .09 | -.25*** | .46*** | -.13* | -.07 |
| 15. Identification with Likeminded People | -.02 | -.07 | .09 | -.07 | -.09 | -.11 |

**Table S3 (cont.)**

| Variable | 7 | 8 | 9 | 10 | 11 | 12 | 13 | 14 | 15 |
| --- | --- | --- | --- | --- | --- | --- | --- | --- | --- |
| 7. Actively Open-Minded Thinking | - |  |  |  |  |  |  |  |  |
| 8. Intellectual Humility | .55*** | - |  |  |  |  |  |  |  |
| 9. Need to Evaluate | -.06 | -.06 | - |  |  |  |  |  |  |
| 10. Bullshit Receptivity | -.28*** | .00 | .12 | - |  |  |  |  |  |
| 11. Conspiracy Mentality | -.23*** | -.04 | .09 | .23*** | - |  |  |  |  |
| 12. Self-Esteem | .03 | -.04 | .06 | .13* | -.04 | - |  |  |  |
| 13. Grandiose Narcissism | -.35*** | -.15* | .30** | .25*** | .21*** | .21*** | - |  |  |
| 14. Need to Belong | -.13* | .01 | .07 | .06 | .02 | -.33*** | -.01 | - |  |
| 15. Identification with Likeminded People | -.20** | -.23*** | .10 | .15* | .03 | .01 | .01 | .02 | - |

Note. * p < .05, ** p < .01, *** p < .001

**Table S4**

*Partial* *Correlations Between Measures Controlling for Political Affiliation (Democrat vs. Republican), Study 1*

| Variable | 1 | 2 | 3 | 4 | 5 | 6 | 7 |
| --- | --- | --- | --- | --- | --- | --- | --- |
| 1. False-Alarm Rate | - |  |  |  |  |  |  |
| 2. Truth Sensitivity | -.30*** | - |  |  |  |  |  |
| 3. Acceptance Threshold | -.83*** | -.25*** | - |  |  |  |  |
| 4. Myside Bias | .09 | -.06 | -.09 | - |  |  |  |
| 5. Extraversion | .05 | -.09 | .01 | -.14* | - |  |  |
| 6. Agreeableness | -.15* | -.04 | .16** | -.02 | .32*** | - |  |
| 7. Conscientiousness | -.10 | -.05 | .13* | -.04 | .43*** | .40*** | - |
| 8. Neuroticism | .12 | -.03 | -.10 | -.03 | -.48*** | -.32*** | -.59*** |
| 9. Openness | -.02 | .02 | -.01 | .14* | .24*** | .20*** | .11 |
| 10. Cognitive Reflection | -.16** | .29*** | .00 | .08 | -.16** | -.05 | -.10 |
| 11. Actively Open-Minded Thinking | -.27*** | .27*** | .11 | .05 | -.12* | .15* | .10 |
| 12. Intellectual Humility | -.12* | .09 | .08 | -.17** | .02 | .09 | -.05 |
| 13. Need to Evaluate | .21*** | -.03 | -.19** | .07 | .22*** | -.13* | -.02 |
| 14. Bullshit Receptivity | .26*** | -.25*** | -.12 | -.22*** | .20*** | .10 | .21*** |
| 15. Conspiracy Mentality | .28*** | -.13* | -.21*** | .07 | .03 | -.17** | -.10 |
| 16. Self-Esteem | -.06 | .06 | .02 | .00 | .52*** | .31*** | .52*** |
| 17. Grandiose Narcissism | .22*** | -.24*** | -.07 | -.06 | .49*** | -.11 | .10 |
| 18. Need to Belong | .06 | -.12* | .01 | -.12 | -.07 | .10 | -.26*** |
| 19. Identification with Likeminded People | .01 | .01 | -.02 | .04 | -.05 | -.06 | .08 |

**Table S4 (cont.)**

| Variable | 8 | 9 | 10 | 11 | 12 | 13 | 14 | 15 | 16 | 17 | 18 | 19 |
| --- | --- | --- | --- | --- | --- | --- | --- | --- | --- | --- | --- | --- |
| 8. Neuroticism | - |  |  |  |  |  |  |  |  |  |  |  |
| 9. Openness | -.11 | - |  |  |  |  |  |  |  |  |  |  |
| 10. Cognitive Reflection | -.01 | -.07 | - |  |  |  |  |  |  |  |  |  |
| 11. Actively Open-Minded Thinking | -.07 | .14* | .25*** | - |  |  |  |  |  |  |  |  |
| 12. Intellectual Humility | -.04 | .15* | .08 | .50*** | - |  |  |  |  |  |  |  |
| 13. Need to Evaluate | .03 | .21*** | -.15 | -.09 | -.09 | - |  |  |  |  |  |  |
| 14. Bullshit Receptivity | -.06 | -.02 | -.25*** | -.24*** | .05 | .13* | - |  |  |  |  |  |
| 15. Conspiracy Mentality | .12* | .06 | -.16** | -.17** | .02 | .12 | .20*** | - |  |  |  |  |
| 16. Self-Esteem | -.71*** | .19** | -.03 | .06 | -.02 | .07 | .12* | -.06 | - |  |  |  |
| 17. Narcissism | -.18** | .06 | -.14* | -.36*** | -.15* | .30*** | .25*** | .20*** | .20*** | - |  |  |
| 18. Need to Belong | .47*** | -.12 | -.06 | -.12 | .03 | .07 | .05 | .00 | -.33*** | -.02 | - |  |
| 19. Identification with Likeminded People | -.06 | -.06 | -.09 | -.15** | -.19** | .12* | .12* | -.01 | .00 | .01 | .01 | - |

Note. * p < .05, ** p < .01, *** p < .001

**Table S5**

*Means and 95% Confidence Intervals of Measures for Participants with Favorable and Unfavorable Attitudes toward COVID-19 Vaccines, Study 2*

|  | Favorable Attitudes | | Unfavorable Attitudes | |
| --- | --- | --- | --- | --- |
|  | *n* = 122 | | *n* = 100 | |
| Variable | *M* | 95% CI | *M* | 95% CI |
| *Misinformation Indices* |  |  |  |  |
| False-Alarm Rate | 0.20 | [0.18, 0.21] | 0.30 | [0.27, 0.32] |
| Truth Sensitivity | 1.59 | [1.49,1.68] | 0.76 | [0.65, 0.87] |
| Acceptance Threshold | 0.11 | [0.08, 0.15] | 0.20 | [0.14, 0.25] |
| Myside Bias | 0.37 | [0.25, 0.50] | 1.54 | [1.34, 1.74] |
| *Individual Difference Measures* |  |  |  |  |
| Extraversion | 2.71 | [2.55, 2.86] | 2.96 | [2.79, 3.14] |
| Agreeableness | 3.95 | [3.83, 4.07] | 3.79 | [3.61, 3.96] |
| Conscientiousness | 3.64 | [3.48, 3.80] | 3.79 | [3.62, 3.95] |
| Neuroticism | 2.67 | [2.49, 2.85] | 2.66 | [2.45, 2.86] |
| Openness | 3.67 | [3.53, 3.81] | 3.78 | [3.62, 3.94] |
| Cognitive Reflection | 4.66 | [4.34, 4.99] | 3.69 | [3.28, 4.10] |
| Actively Open-Minded Thinking | 4.63 | [4.53, 4.73] | 4.39 | [4.28, 4.51] |
| Intellectual Humility | 3.95 | [3.84, 4.05] | 3.93 | [3.78, 4.07] |
| Need to Evaluate | 2.84 | [2.72, 2.96] | 3.16 | [3.02, 3.30] |
| Bullshit Receptivity | 2.38 | [2.23, 2.53] | 2.69 | [2.52, 2.87] |
| Conspiracy Mentality | 5.53 | [5.22, 5.84] | 7.70 | [7.36, 8.04] |
| Self-Esteem | 3.00 | [2.89, 3.11] | 3.03 | [2.90, 3.16] |
| Grandiose Narcissism | 1.76 | [1.38, 2.14] | 3.25 | [2.65, 3.85] |
| Need to Belong | 2.83 | [2.71, 2.95] | 2.62 | [2.45, 2.79] |
| Identification with Likeminded People | 0.16 | [0.05, 0.27] | 0.36 | [0.20, 0.51] |

**Table S6**

*Correlations Between Individual-Difference Measures and Ideology-Congruent and Ideology-Incongruent False-Alarm Rates, Study 1*

| Variable | Ideology-Congruent False Alarm Rate | Ideology-Incongruent False Alarm Rate |
| --- | --- | --- |
| Extraversion | .12 | -.09 |
| Agreeableness | -.04 | .06 |
| Conscientiousness | .09 | .02 |
| Neuroticism | .04 | .04 |
| Openness | .07 | -.08 |
| Cognitive Reflection | -.24*** | -.07 |
| Actively Open-Minded Thinking | -.27*** | -.18** |
| Intellectual Humility | -.05 | .07 |
| Need to Evaluate | .23*** | .00 |
| Bullshit Receptivity | .14 | .21** |
| Conspiracy Mentality | .38*** | .04 |
| Self-Esteem | .02 | -.09 |
| Grandiose Narcissism | .20** | -.01 |
| Need to Belong | -.13 | .06 |
| Identification with Likeminded People | .06 | .00 |

Note. * p < .05, ** p < .01, *** p < .001

**Table S7**

*Correlations Between Individual-Difference Measures, Study 2*

| Variable | 1 | 2 | 3 | 4 | 5 | 6 |
| --- | --- | --- | --- | --- | --- | --- |
| 1. Extraversion | - |  |  |  |  |  |
| 2. Agreeableness | .13 | - |  |  |  |  |
| 3. Conscientiousness | .46*** | .32*** | - |  |  |  |
| 4. Neuroticism | -.44*** | -.25*** | -.42*** | - |  |  |
| 5. Openness | .17* | .13 | .07 | -.19** | - |  |
| 6. Cognitive Reflection | -.06 | -.06 | -.16* | -.08 | .09 | - |
| 7. Actively Open-Minded Thinking | -.13 | .12 | -.08 | -.16* | .11 | .29*** |
| 8. Intellectual Humility | -.11 | .23*** | -.16* | .03 | .10 | .16* |
| 9. Need to Evaluate | .23*** | -.11 | .10 | -.02 | .18** | -.11 |
| 10. Bullshit Receptivity | .21** | .16* | .20** | -.01 | -.01 | -.35*** |
| 11. Conspiracy Mentality | .15* | -.05 | .04 | .04 | .03 | -.29*** |
| 12. Self-Esteem | .53*** | .29*** | .43*** | -.71*** | .22** | .08 |
| 13. Grandiose Narcissism | .51*** | -.24*** | .12 | -.26*** | .13 | -.06 |
| 14. Need to Belong | -.06 | .21** | .01 | .33*** | -.07 | -.07 |
| 15. Identification with Likeminded People | .06 | -.12 | .09 | .12 | -.05 | -.06 |

**Table S7 (cont.)**

| Variable | 7 | 8 | 9 | 10 | 11 | 12 | 13 | 14 | 15 |
| --- | --- | --- | --- | --- | --- | --- | --- | --- | --- |
| 7. Actively Open-Minded Thinking | - |  |  |  |  |  |  |  |  |
| 8. Intellectual Humility | .51*** | - |  |  |  |  |  |  |  |
| 9. Need to Evaluate | -.14* | -.16* | - |  |  |  |  |  |  |
| 10. Bullshit Receptivity | -.26*** | .11 | .15* | - |  |  |  |  |  |
| 11. Conspiracy Mentality | -.28*** | .03 | .30*** | .23*** | - |  |  |  |  |
| 12. Self-Esteem | .09 | -.10 | .05 | .04 | .02 | - |  |  |  |
| 13. Narcissism | -.13 | -.13 | .24*** | .05 | .26*** | .29*** | - |  |  |
| 14. Need to Belong | -.09 | -.04 | .16* | .09 | -.07 | -.25*** | -.20** | - |  |
| 15. Identification with Likeminded People | -.22*** | -.29*** | .14* | .13* | .06 | -.09 | .11 | .06 | - |

Note. * p < .05, ** p < .01, *** p < .001

**Table S8**

*Partial* *Correlations Between Measures Controlling for COVID-19 Vaccine Attitudes (Favorable vs. Unfavorable), Study 2*

| Variable | 1 | 2 | 3 | 4 | 5 | 6 | 7 |
| --- | --- | --- | --- | --- | --- | --- | --- |
| 1. False-Alarm Rate | - |  |  |  |  |  |  |
| 2. Truth Sensitivity | -.79*** | - |  |  |  |  |  |
| 3. Acceptance Threshold | -.67*** | .11 | - |  |  |  |  |
| 4. Myside Bias | .31*** | -.37*** | -.07 | - |  |  |  |
| 5. Extraversion | .01 | -.03 | .01 | .05 | - |  |  |
| 6. Agreeableness | .03 | .02 | -.05 | -.04 | .15* | - |  |
| 7. Conscientiousness | .07 | -.06 | -.02 | -.00 | .45*** | .33*** | - |
| 8. Neuroticism | .07 | -.08 | -.02 | .02 | -.44*** | -.26*** | -.43*** |
| 9. Openness | .00 | .03 | .01 | .05 | .16* | .13* | .06 |
| 10. Cognitive Reflection | -.19** | .23*** | .03 | .02 | -.02 | -.09 | -.14* |
| 11. Actively Open-Minded Thinking | -.31*** | .31*** | .12 | -.01 | -.10 | .10 | -.07 |
| 12. Intellectual Humility | .00 | .07 | -.08 | -.08 | -.11 | .23*** | -.16* |
| 13. Need to Evaluate | .15* | -.13 | -.07 | .03 | .20** | -.08 | .08 |
| 14. Bullshit Receptivity | .19** | -.14* | -.11 | -.15* | .19** | .18** | .19** |
| 15. Conspiracy Mentality | .22*** | -.23*** | -.04 | -.05 | .08 | .01 | -.01 |
| 16. Self-Esteem | -.04 | .02 | .06 | .06 | .53*** | .30*** | .43*** |
| 17. Grandiose Narcissism | .09 | -.09 | -.03 | .00 | .50*** | -.22*** | .10 |
| 18. Need to Belong | -.04 | .05 | .03 | -.10 | -.04 | .19** | .03 |
| 19. Identification with Likeminded People | .00 | -.01 | .01 | -.05 | .04 | -.10 | .08 |

**Table S8 (cont.)**

| Variable | 8 | 9 | 10 | 11 | 12 | 13 | 14 | 15 | 16 | 17 | 18 | 19 |
| --- | --- | --- | --- | --- | --- | --- | --- | --- | --- | --- | --- | --- |
| 8. Neuroticism | - |  |  |  |  |  |  |  |  |  |  |  |
| 9. Openness | -.19** | - |  |  |  |  |  |  |  |  |  |  |
| 10. Cognitive Reflection | -.08 | .11 | - |  |  |  |  |  |  |  |  |  |
| 11. Actively Open-Minded Thinking | -.17* | .12 | .25*** | - |  |  |  |  |  |  |  |  |
| 12. Intellectual Humility | .03 | .10 | .16* | .51*** | - |  |  |  |  |  |  |  |
| 13. Need to Evaluate | -.02 | .17* | -.05 | -.10 | -.16* | - |  |  |  |  |  |  |
| 14. Bullshit Receptivity | -.01 | -.02 | -.32*** | -.23*** | .11 | .11 | - |  |  |  |  |  |
| 15. Conspiracy Mentality | .05 | -.01 | -.19** | -.21** | .04 | .21** | .16** | - |  |  |  |  |
| 16. Self-Esteem | -.71*** | .22*** | .09 | .10 | -.10 | .04 | .03 | .01 | - |  |  |  |
| 17. Grandiose Narcissism | -.27*** | .11 | .01 | -.07 | -.13 | .19** | .00 | .14* | .29*** | - |  |  |
| 18. Need to Belong | .33*** | -.06 | -.11 | -.12 | -.04 | .20** | .12 | .01 | -.25*** | -.17* | - |  |
| 19. Identification with Likeminded People | .12 | -.06 | -.03 | -0.20** | -.29*** | .12 | .11 | -.03 | -.10 | .08 | .08 | - |

Note. * p < .05, ** p < .01, *** p < .001

**Table S9**

*Correlations between SDT Indices and Demographic Measures*

| Variable | Truth  Sensitivity | Acceptance Threshold | Myside  Bias |
| --- | --- | --- | --- |
| *Study 1* |  |  |  |
| Political Orientation ^a^ | -.09 | -.04 | -.16** |
| Political Interest | .15* | -.11 | .24** |
| Social Media Use | -.06 | .04 | -.02 |
| Age | .16** | -.04 | .07 |
| Gender ^b^ | .10 | .03 | -.02 |
| Education | .01 | -.06 | -.15* |
| *Study 2* |  |  |  |
| Political Orientation ^a^ | -.33*** | .06 | .22*** |
| Political Interest | .09 | -.02 | .03 |
| Social Media Use | -.07 | .04 | .08 |
| Age | .05 | -.04 | -.05 |
| Gender ^b^ | .03 | -.08 | -.05 |
| Education | .15* | -.01 | .02 |

*Note.* ^a^ Higher scores on the measure of political orientation indicate a stronger conservative (vs. liberal) political orientation; ^b^ Positive correlations indicate higher scores among women (vs. men). * *p* < .05, ** *p* < .01, *** *p* < .001.

1. To calculate ω_H_, bullshit receptivity and conspiracy mentality items were reverse-coded, so that higher scores on all items contribute to higher composite scores on the general factor. [↑](#footnote-ref-2)
